# A Comparative Study of Pathology and Host Immune Response Induced by Very Virulent Infectious Bursal Disease Virus in Experimentally Infected Chickens of Aseel and White Leghorn Breeds

**DOI:** 10.3390/vaccines8040627

**Published:** 2020-10-26

**Authors:** Shyama N. Prabhu, Ajay Pratap Singh, Berin P. Varghese, Kuldeep Dhama, Shambhu Dayal Singh, Rajendra Singh

**Affiliations:** 1 Department of Veterinary Pathology, College of Veterinary Sciences and Animal Husbandry, Deen Dayal Upadhayay Pashu Chikitsa Vigyan Vishwavidyalay Evum Go-Anusandhan Sansthan (DUVASU), Mathura UP 281001, India; 2 Department of Veterinary Microbiology, College of Veterinary Sciences and Animal Husbandry, Deen Dayal Upadhayay Pashu Chikitsa Vigyan Vishwavidyalay Evum Go-Anusandhan Sansthan (DUVASU), Mathura UP 281001, India; drajaysinghvet2001@gmail.com; 3 The Avrum Gudelsky Veterinary Center, College Park, University of Maryland, College Park, MD 20742, USA; berin05@gmail.com; 4 Division of Pathology, ICAR-Indian Veterinary Research Institute, Izatnagar, Bareilly UP 243 122, India; kdhama@rediffmail.com (K.D.); sdsingh2005@gmail.com (S.D.S.)

**Keywords:** infectious bursal disease, pathology, immune response

## Abstract

Indigenous breeds of young chickens in India are believed to be resistant to the classical strain of infectious bursal disease virus (IBDV). However, the mechanism underlying this resistance is obscure. Innate immunity is a key factor in defining the clinical course and pathology of microbial infections. The present study is aimed to compare the pathology of very virulent IBDV (vvIBDV) and immunological host response in experimentally infected - vaccinated and unvaccinated indigenous Aseel and commercial White Leghorn chickens. The viral loads and innate immune gene expression profiles of MDA-5, Mx, IFN-α, and IFN-β in different lymphoid organs were analyzed by quantitative PCR. The histopathological scores in Aseel birds were lower than in White Leghorns despite comparable viral loads. The degrees of histopathological lesions were fewer in vaccinated birds than in unvaccinated birds of both breeds. Analysis of innate immune response genes revealed that the cytoplasmic pattern recognition receptor MDA-5 gene was overexpressed mainly in the cecal tonsils of both vaccinated and nonvaccinated White Leghorn chickens. An increase in the expression of the IFN-α gene was seen in the cecal tonsils of Aseels, and an increase in IFN-β gene expression was seen in the thymuses of White Leghorns following vvIBDV challenge both in vaccinated and nonvaccinated birds. In addition, we observed that the Mx gene plays a minimal role, if any, in vvIBDV infection of the breeds under study. It remains interesting and important that although vvIBDV causes disease in indigenous Aseel birds, the faster clearance and reduced pathology of the virus in Aseel birds compared to White Leghorn chicken indicate some unidentified innate immune factors that are limiting IBDV in this breed. Further studies will be required to correlate kinetics of humoral and cellular immune response in relation to the virus load in different organs to illuminate the mechanism of genetic resistance in native breeds of chicken.

## 1. Introduction

Infectious bursal disease (IBD) is one of the most important emerging viral diseases in the poultry industry. It is caused by a non-enveloped virus belonging to the family *Birnaviridae* with a bisegmented double-stranded RNA (dsRNA) genome [1]. The virus has a predilection for pre-B lymphocytes in the bursa of Fabricius. Birds at 3–6 weeks of age display clinical signs, principally when the bursa is at its maximum development and when maternal antibodies are waning [2]. The disease is economically important because of the high mortality rates attributed to a highly virulent emergent variant of the virus capable of infecting vaccinated chickens and causing severe immunosuppression in surviving birds. However, mechanisms of pathogenic and immunosuppressive aspects of IBDV are still not well known [3].

Chickens of all breeds are susceptible to infectious bursal disease virus (IBDV) infection [4]. Lighter breeds of chickens such as the White Leghorn (WLH) are considered more susceptible than heavier breeds [5,6]. In addition, layer-type chickens of all genetic backgrounds show higher IBD viral antigenic loads in the bursa compared to the broiler breeds [7]. Broiler breeds were also found to be less susceptible to IBDV based on an assessment of clinical signs and lesions [8]. Aseel breed of chicken is considered a native chicken breed and therefore believed to be resistant to the IBD. The resistance of native breeds to tropical diseases is thought to be specific to genetic line and disease, mainly because of their natural selection in tropical environments.

Both innate and adaptive immunities are required for highly efficient recognition and clearance of infective pathogens. Highly conserved components of the innate immune system are key factors in determining the clinical course and pathology of microbial infections. This immune arm provides the first line of defense against microbial pathogens [9,10,11]. The excessive or insufficient production of innate immune factors such as pro-inflammatory cytokines determines the pathophysiology of the disease by regulating inflammation and immunity [12].

Melanoma differentiation-associated gene-5 (MDA-5), an intracytoplasmic pattern recognition receptor, belongs to the retinoic acid-inducible gene I (RIG-I)-like receptor (RLR) family [13]. MDA-5 is said to recognize long dsRNA independent of its phosphorylation status [14]. Once MDA-5 binds with viral dsRNA, it can make active a number of downstream transcription factors that translocate to the nucleus and stimulate expression of type I interferon (IFN) genes [15,16,17]. The type-I IFN pathway plays a central role in innate immune responses to viral infection, inducing expression of multiple genes encoding kinases such as 2′, 5′oligoadenylatesynthatase and the myxoma gene (Mx), which is known to encode proteins that participate in antiviral responses [18,19]. Mx is one of the best-characterized interferon-regulated antiviral genes [20,21], and it has shown antiviral activity against various RNA viruses through post-transcriptional regulation and interaction with nucleocapsid-like structures, which restricts the localization of certain viruses within cells [22,23].

Although the critical role of MDA-5 in the recognition of several RNA viruses [24,25] and its effects on chicken embryo fibroblasts [26] have been shown, its role in IBDV infection in live birds is yet to be determined. Because of this gap, the relative expression of innate immune response genes, namely MDA-5, Mx, IFN-α, and IFN-β in the bursa, spleen, thymus, and cecal tonsils of chickens was determined using real-time PCR (RT-PCR). Absolute quantification of vvIBDV in these lymphoid organs in both vaccinated and nonvaccinated challenged WLH and Aseel chickens was also performed. The indigenous Aseel breeds showed minimal changes in various lymphoid organs compared to the commercial white leghorn breed despite comparable virus load. IFN-α was found to be up-regulated in Aseel breeds whereas higher gene expression of IFN-β was observed in WLH. No significant increase in expression of the Mx gene was seen in any of the experimental groups studied. Our results define an essential role for Interferons (IFN-α IFN-β) and demonstrate redundant roles for MDA-5 and Mx genes involved in the innate immune response to vvIBDV infection in different chicken breeds.

## 2. Materials and Methods

### 2.1. Source of Virus

The very virulent IBDV (strain UP-01-2013), maintained in the Avian Viral Diseases Lab, Division of Pathology, Indian Veterinary Research Institute, Bareilly, India, was used to prepare the inoculums used in the challenge study. The virus was propagated in 10-day-old specific-pathogen-free (SPF) embryonated chicken eggs by the chorioallantoic membrane (CAM) route, using the method described by [27]. The stock virus was quantified by determining the 50% egg infectious dose (EID_50_) [28].

### 2.2. Experimental Birds

One-day-old Aseel (*n* = 54) and WLH (*n* = 54) chickens were divided randomly into six groups of 18 birds each and housed individually under strict hygienic conditions. The seronegativity of these chicks for IBDV was confirmed by commercial antibody enzyme-linked immunosorbent assay kit for IBD (IDEXX Laboratories, Inc., Westbrook, ME, USA). The experiment was carried out after approval from the Institute Animal Ethics Committee of the Indian Veterinary Research Institute (Approval no. F.1-53/2012-13/JD-R).

### 2.3. Experimental Design

The experimental birds were divided into six groups: (1) NWG, consisting of nonvaccinated challenged WLH; (2) VWG, consisting of vaccinated challenged WLH; (3) CWG, which served as a WLH control and were neither vaccinated nor challenged; (4) NAG, consisting of nonvaccinated challenged Aseel; (5) VAG, consisting of vaccinated challenged Aseel, and (6) CAG, which served as Aseel controls and were neither vaccinated nor challenged. At 2 weeks of age, chickens in the VWG and VAG groups were vaccinated with a single dose of Georgia strain Infectious Bursal Disease (Gumboro) Live Vaccine (Indovax Pvt. Ltd., India). At 4 weeks of age, chickens in the four groups, namely NWG, VWG, NAG, and VAG, were inoculated with 0.5 mL/chick of the vvIBDV inoculum with a titer of 10^5^ egg infectious dose (EID_50_)/mL through the oral route. Chicks from both of the control groups, i.e., CWG and CAG, were sham inoculated with 0.5 mL of virus diluents. The birds were monitored for clinical signs up to 5 days post-inoculation (dpi). Three birds from each group were sacrificed at 24 h post-inoculation and successively up to 5 days at 24 h intervals. Gross pathological lesions in different organs were noted. Based on gross lesions in different lymphoid organs namely bursa, spleen, thymus and caecal tonsils scoring were done. Samples of lymphoid organs, namely the bursa, spleen, thymus, and cecal tonsils, were collected in RNA Later (Sigma, USA) for preservation at −20 °C until absolute quantification of viral load and relative quantification of immune response genes MDA-5, IFN-α, IFN-β, and Mx genes by RT-PCR. Remaining bursa, spleen, thymus, cecal tonsil, and Harderian gland (HG) tissues were collected in 10% neutral buffered formalin for histopathology.

### 2.4. Histopathology

Representative tissues were routinely processed for paraffin embedding and 5–6-µm thick sections were cut. Routine hematoxylin and eosin (H&E) staining was performed as per the conventional procedure. Lesions in spleens, thymuses, and cecal tonsils were scored according to the method proposed by earlier workers [29] with some modifications. Bursal lesions were scored according to the method of Williams and Davison [30] with slight modifications.

### 2.5. Immunohistochemistry

For immunohistochemistry (IHC), 5–6-µm thick sections were cut from paraffin-embedded tissue blocks on Poly-l-lysine (Sigma-Aldrich, St. Louis, MO, USA)-coated slides. Bursal sections were deparaffinized by baking in an oven at 60 °C for 1 h and given two washes of xylene (10 min each). The sections were rehydrated in descending grades of alcohol to remove the xylene, followed by 95%, 70%, and 50% alcohol. The sections were then submerged in phosphate-buffered saline (PBS) (pH 7.4). Heat-induced antigen retrieval was performed by microwaving the sections in Coplin jars containing 10 mM Tri-sodium citrate buffer (pH 6) for 20 min. The slides were incubated in 0.3–3% H_2_O_2_ in absolute methanol for 15 min in the dark to quench endogenous peroxidase activity. Non-specific antigen blocking was done by covering the sections with 5% normal goat serum in PBS (pH 7.4). Immunostaining was performed by adding primary antibody, raised in rabbits against vvIBDV, at a dilution of 1:20. Goat Anti-Rabbit IgG HRPO (Cat no.105499, Genei) was used as the secondary antibody at the dilution rate of 1:40 in PBS. The titer of primary antibody raised in rabbits against vvIBDV was checked by Indirect haemaglutination test (IHA) and found to be Log_2_^8^. 3-Amino-9-ethylcarbazole (product no. AEC 101, Sigma-Aldrich) substrate was used according to the manufacturer’s protocol, and then sections were counterstained with Mayer’s hematoxylin for 2 to 3 min. The specificity of staining was confirmed by excluding primary antibodies in the controls. The degree or intensity of vvIBDV infection was scored as described by earlier workers [31]. IHC scoring was based on the percentage of antigen-positive cells in 10 randomly selected high-power fields.

### 2.6. RNA Preparation

Total RNA was extracted from lymphoid organs using TRIzol (Thermo Fisher Scientific, Waltham, MA, USA) as per the manufacturer’s protocol. Isolated total RNA was treated with deoxyribonuclease-1 to avoid DNA contamination using AMPD-1 kit (product no. AMPD1-1kt, Sigma-Aldrich, St. Louis, MO, USA). The purity of RNA was checked by measuring the optical density value using a NanoDrop 1000 (Thermo Fisher Scientific, USA) at wavelengths of 260 and 280 nm. Samples with a 260/280 ratio of 1.8 or above were selected for cDNA synthesis. Reverse transcription for the first-strand synthesis was done by using a standard reverse transcription procedure and RevertAid First Strand cDNA Synthesis Kit (Thermo Fisher Scientific, USA). The cDNA samples were cooled at 4 °C and stored at −20 °C.

### 2.7. Quantitative Real-Time RT-PCR

The real-time PCR for relative quantification of chicken MDA-5, IFN-α, IFN-β, Mx, and GAPDH genes was performed using SYBR Green Real-Time Master Mix (Thermo Fisher Scientific, San Jose, CA, USA), respective primer sets (Table 1), and a QB 96 Server Gradient Thermal Cycler (Quanta Biotech Ltd., Surrey, UK). A reaction mixture containing 10 μL SYBR Green Real-Time Master Mix, 1.0 μL of a 10-pmole working solution of each gene-specific forward and reverse primer, 1.0 μLof cDNA, and RNase-free water up to a final volume of 20 μL, was subjected to the following thermal cycle: one cycle of 95 °C for 10 min and 40 cycles of 95 °C for 15 s, 60 °C for 30 s, and 72 °C for 30 s. A dissociation curve analysis after real-time amplification was performed at 95 °C for 1 min, 61 °C for 30 s, and 95 °C for 30 s. To determine the relative change in gene expression, the 2^^−^^∆∆Ct^ method was employed [32]. Then, the following formula was used to calculate the fold change in gene expression: fold change = 2^^−^^∆∆Ct^, where ∆∆Ct = (Ct of the target gene – Ct of GAPDH) treatment – (Ct of the target gene – Ct of GAPDH) control. The results of the gene-specific amplification were corrected for the difference in the quantity of input RNA by comparison with expression levels of the housekeeping gene GAPDH, and the results were analyzed by one-way analysis of variance (ANOVA) to determine statistically significant differences between mean values of each group. Values with *p* < 0.05 were considered significant.

### 2.8. Determination of Viral Load and Preparation of a Standard Curve

**Figure A1:**
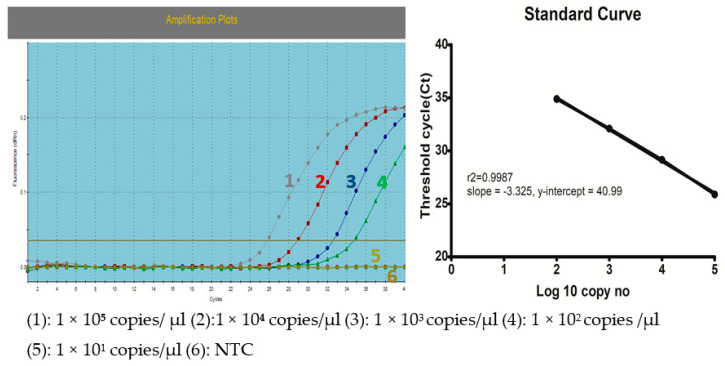
Amplification plot and Standard curve for quantitation of vvIBDV copy number by real-time PCR. Q-PCR performed on serial ten-fold dilution from 1.0 × 105 to 1 × 101 copies/µl with NTC as control. Standard curve with Log10 copy number plotted as X-axis and Ct values on Y-axis. Slope = −3.325; y-intercept = 40.99.

The cDNA was synthesized using a standard reverse transcription procedure and cDNA synthesis kit. RT-PCR was performed with a known positive vvIBDV sample using the following primer pair: forward, 5′-AGATAACCCAGCCAATCAC-3, and reverse, 5′-CACTCTTTCGTAAGCTACTAGTG-3′. The amplified product was purified using a QIAquick gel extraction kit (Qiagen). The mean DNA concentration of each purified fragment was determined by NanoDrop A260 measurement, and the number of molecules of construct per microliter was determined. The copy number of the 172-bp segment was calculated according to a method described earlier [37]. To generate the standard curve, 10-fold serial dilutions of the amplification product having 10^5^ to 10^1^ copies of amplified DNA were prepared in triplicate along with non-template controls for RT-PCR with SYBR Green Master Mix in a QB 96 Server Gradient Thermal Cycler (Quanta Biotech Ltd., UK). The PCR mixtures (20 µL) contained 10 μL SYBR Green Real-Time Master Mix, 1.0 μL of a 10-pmole working solution of each gene-specific forward and reverse primer for vvIBDV, 1.0 μL of cDNA, and RNase-free water up to a final volume of 20 μL. The thermal cycling conditions comprised an initial heat-denaturing step at 95 °C for 10 min, followed by 40 cycles of amplification, each with denaturation at 95 °C for 15 s, annealing at 56 °C for 15 s, and extension at 72 °C for 15 s. The log_10_ copy number in each dilution was plotted against the Ct value obtained to generate a standard curve. Viral copy numbers for each sample were interpolated from the standard curve by linear regression. The amplification plot and standard curve for quantitation of vvIBDV copy number by real-time PCR has been provided as additional image (Appendix A, Figure A1). In the regression analysis of the Ct values generated from the dilution series, the following values were obtained: correlation coefficient, 0.9987; amplification factor, 2.036; and PCR efficiency, 103.5%. The slopes of the regression lines were −3.235. The limit of detection (LOD) of the vvIBDV genome was 10^2^ copies/µl.

### 2.9. Statistical Analyses

Data were analyzed by using analysis of variance (ANOVA) with GraphPad Prism 5 software (Graphpad Software Inc., San Diego, CA, USA). Post hoc tests were calculated using the Bonferroni’s multiple comparison tests. Results were designated significant when the *p*-value *p* < 0.05 *, *p* < 0.01 **, *p* < 0.001 ***.

## 3. Results and Discussion

**Figure 1:**
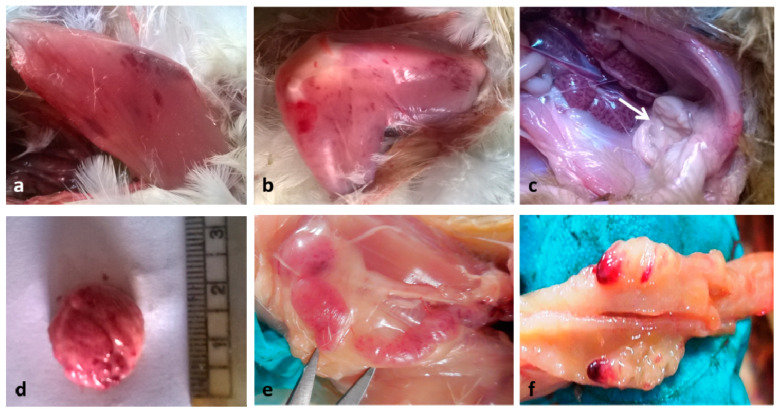
Gross pathological lesions caused by the vvIBD virus in different groups of chicken. (**a**) Paintbrush pattern hemorrhages in the breast and (**b**) thigh muscles of NWG at 4 dpi. (**c**) Edematous enlarged bursa (arrow) and enlarged pale kidneys with tubules distended with urates in NAG at 4 dpi. (**d**) Enlarged and hemorrhagic bursa in NWG on 5 dpi. (**e**) Petechial hemorrhages in the thymus of NWG at 5 dpi. (**f**) Hemorrhages in caecal tonsils of NAG at 3 dpi. NWG- Nonvaccinated challenged White Leghorn Group; NAG-Nonvaccinated challenged Aseel Group.

In the present study, the challenged birds showed reluctance to move when provoked, ruffled feathers, anorexia, and white watery diarrhea. The clinical signs in infected Aseel chicks were less intense than those in WLH. This agrees with the finding that vvIBDV does not induce overt clinical signs in three-week-old Aseel chickens, whereas signs are apparent in WLH of the same age [38]. However, no mortality was recorded in any group during the study period. This may be because recorded deaths have been associated with various factors, including the route of inoculation, viral dose, age of birds, and the presence of other poultry pathogens [39]. Gross lesions such as hemorrhage in the breast and thigh muscles, enlargement and edematous bursa, renal changes, and hemorrhage in cecal tonsils were noted (Figure 1), except in vaccinated Aseel group, in which gross lesions were negligible. The gross lesions seen in nonvaccinated Aseel were of less intensity than those in nonvaccinated WLH. The lesions encountered were consistent with those reported previously [40,41]. A semi-quantitative assessment of gross pathology among the challenged and control birds is shown in Table 2.

**Figure 2:**
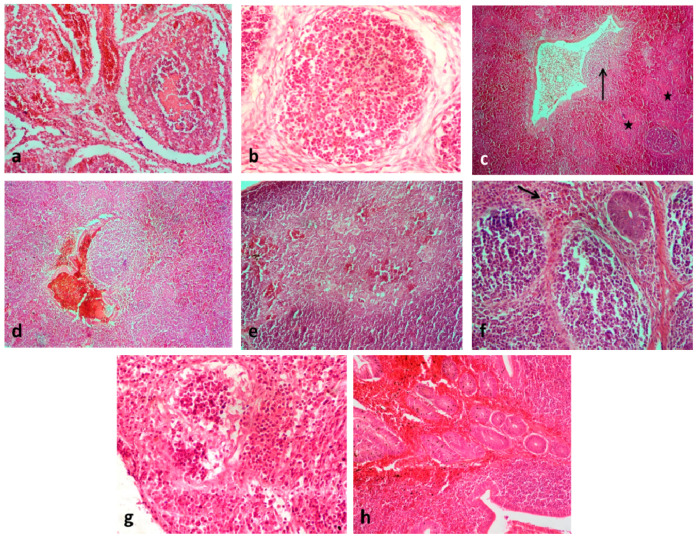
Histopathological lesions in lymphoid organs of different groups. (**a**) Bursa of NWG with depletion of lymphocytes, cystic cavities in the medulla, extensive hemorrhages and disruption of bursal architecture at 5 dpi H&EX200. (**b**) Bursa of NAG showing rounding of follicles, depletion of lymphocytes and increased macrophages and fibroplasia at 5 dpi H&EX400. (**c**) The spleen of NWG showing areas of necrosis (*) and mild depletion of lymphoid cells around the trabecular vein (→) at 2 dpi and (**d**) hemorrhages at 5 dpi H&EX100. (**e**) Hemorrhages in the thymus of NWG at 1 dpi H&EX200. (**f**) Caecal tonsils showing depletion of lymphocytes and infiltration of heterophils (→) in NWG at 1 dpi. (**g**) Caecal tonsils showing severe depletion of lymphocytes and an increase in macrophages (→) in NWG at 3 dpi. H&EX400. (**h**) Hemorrhages in caecal tonsils of NAG at 3 dpi H&EX100.

Comparative histopathology of different lymphoid organs showed that the lesions in bursae of vaccinated WLH, nonvaccinated WLH, and nonvaccinated Aseel chickens were comparable at all time points in the study, except at 5 dpi when lesions were most severe in the nonvaccinated WLH group (Figure 2a and Table 3). Chickens in the nonvaccinated Aseel group showed rounding of follicles, depletion of lymphocytes, and elevated numbers of macrophages with increased fibroplasia (Figure 2b). However, chickens in the vaccinated Aseel group showed relatively normal bursal architecture. There are reports [42] suggesting a significantly higher mean Bursa Body Weight Ratio (BBWR) in nonvaccinated challenged WLH than the control birds, while the pre-infection bursal index of WLH was reported greater than Aseel birds. The spleens from chickens in the vaccinated Aseel group were nearly normal throughout the study. Reactive splenic white pulp with increased numbers of germinal centers were seen at 3, 4, and 5 dpi in the vaccinated group. Mild depletion of lymphocytes, with focal areas of necrosis, as seen in the splenic parenchyma of unvaccinated challenged WLH (Figure 2c). Focal areas of hemorrhage were also encountered (Figure 2d). In a previous study [43], a similar increase in germinal centers in vaccinated birds, and splenic necrosis in nonvaccinated IBDV-infected birds was observed.

The thymus of chickens in the Nonvaccinated WLH showed focal hemorrhages and mild cortical lymphocyte depletion (Figure 2e). A significant difference was seen in the histopathological scores of the thymus in both the groups of white leghorns at 3rd, 4th and 5th dpi as compared to the control. However, the histopathological scores of the thymus in both groups of Aseel were close to normal at all study intervals (Table 3). The cecal tonsils of vaccinated Aseel chickens were comparatively normal. Depletion of lymphocytes from a few follicles and aggregated lymphoid tissue with an increase in macrophages (Figure 2f,g) were common findings in cecal tonsils of chickens in the remaining three groups. Hemorrhages in cecal tonsils were also observed in NAG (Figure 2h). Histopathological scores in cecal tonsils were more on 1st, 2nd, and 3rd dpi in NAG with a gradual decrease thereafter. Histopathological scores in the different groups are presented in Table 3.

**Figure 3:**
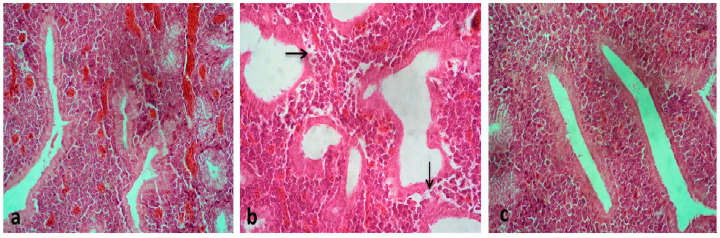
(**a**) Harderian gland of VWG showing hyperemia at 1 dpi. (**b**) HG of NWG showing hyperemia, necrosis and mild depletion of plasma cells (arrows) at 2 dpi. (**c**) HG of control birds densely populated with plasma cells H&E X400.

Avian Harderian Gland (HG) is thought to have a good vascular supply that favors the spread of infectious agents throughout the organ. Avian HG is comprised of three types of lymphoid cells namely, heterophils, lymphocytes, and plasma cells and become densely populated with mainly the plasma cells when the birds reach about 3–4 weeks of age [44]. Hyperemia in HGs was observed in both the groups of White Leghorn at 1st and 2nd dpi (Figure 3a). Necrosis and mild depletion of plasma cells were observed from 2dpi in all the birds of non-vaccinated white leghorn groups but not in the vaccinated group (Figure 3b). However, only a single isolated case of HG hyperemia was noticed at 1dpi in an Aseel bird from NAG. In contrast, HGs of the control birds were densely populated with plasma cells (Figure 3c). The reduction in plasma cells, however, is considered transient, with numbers returning to normal after 14 days [45]. Microscopic alteration in HG namely necrosis, depletion of plasma cells and degeneration of glandular epithelial cells has been reported earlier by many workers [46,47]. Necrosis and the depletion of plasma cells may be the result of direct viral activity.

**Figure 4:**
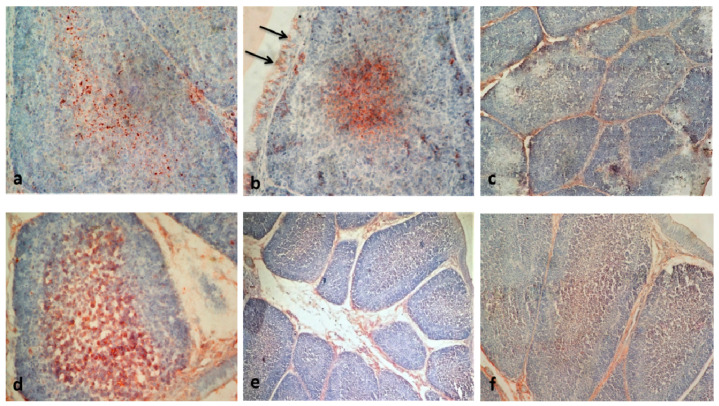
Immunohistochemical staining of bursa detecting the vvIBD viral antigen by IPO-AEC- Mayer’s Haematoxylin. (**a**) Bursa of VWG showing vvIBD viral antigen in medullary and a few cortical cells at 1 dpi. IHC 400X. (**b**) The positive staining could also be seen in the cytoplasm of follicle associated epithelial cells (→), a few cortical and medullary cells in NWG at 1 dpi. IHC 400X. (**c**) Bursa of CWG showing no staining for the virus. IHC 100X. (**d**) A bursal follicle of NAG showing intense staining as well as lymphoid depletion at 3 dpi. IHC 400X. (**e**) Bursa from NAG (where the omission of primary antibodies was done) showing a significant widening of interfollicular spaces but no staining for the virus at 1dpi. IHC 100X and (**f**) bursa of CAG showing no staining for the virus. IHC 200X.

Immunohistochemical reactions to vvIBDV antigens in bursal tissues were assayed and graded in the present study. The Immunohistochemistry (IHC) results showed that viral antigen was present in chickens in all the four experimental groups (Table 4). An important finding in our study was that the localization of the vvIBDV antigen was primarily in the medulla, some cortical cells, and in the cytoplasm of follicle-associated epithelial cells (Figure 4a,b). Similar findings were observed in earlier studies [30]. Viral replication results in severe lymphoid cell damage in medullary and cortical areas of follicles [48]. Intense staining of bursal follicles with lymphoid depletion and interfollicular edema was seen from 2 dpi onwards in VWG, NWG, and NAG (Figure 4d). Positive IHC staining in bursal tissues of vaccinated Aseel chickens indicated that viral antigen was present in bursae but was not associated with significant lesions. To check the specificity of staining, the primary antibody was omitted in the testing of the positive control (Figure 4e). Bursas from the control White Leghorn and control Aseel were kept as the negative controls (Figure 4c,f). All the control groups showed no staining for the virus.

Viral loads in various lymphoid organs of vaccinated and nonvaccinated challenged birds with vvIBDV are presented in Table 5.

In vaccinated WLH chickens, significant viral replication occurred in all the organs except CT (*p* < 0.01). T-cells play an important role in IBD virus replication in various lymphoid organs. Vaccination with Intermediate IBDV strain has known to cause significant thymus atrophy due to the migration of vaccine virus from the spleen to the thymus during 15-day post-vaccination and consequent depletion of T cells [49]. Kim et al. [50] have shown that T cell-intact birds had a significantly lower IBDV-antigen load than T cell-depleted-birds. The significant higher viral load in vaccinated WLH might be due to vaccine-induced T-cells depletion. A similar study of vaccinated SPF chickens conducted by some researchers earlier [51] showed virus replication in chickens challenged one week after vaccination in comparison to those challenged three weeks after vaccination. After vaccination, it takes some time to develop an optimal immune response. Challenge with vvIBDV, two weeks after vaccination might have resulted in viral replication in the bursa of chickens in the vaccinated WLH group. In both vaccinated and nonvaccinated Aseel birds, no significant virus load could be observed in any of the organs barring exceptionally high virus load in the bursa of NAG at 2 dpi (*p* < 0.001). Almost no histopathological lesions and undetectable virus in vaccinated challenged Aseel showed that the vaccine might have had a protective effect in this breed. Previous immune sensitization through vaccination might result in the attenuation of secondary viral replication.

Viral loads were higher in nonvaccinated WLH than in nonvaccinated Aseel birds in all organs at all-time points, except for bursae, with viral loads that were higher in nonvaccinated Aseel group than in nonvaccinated WLH on 2 dpi. In a previous study of SPF chickens, the highest viral load in most organs was detected on 2 dpi [52]. The significant high (*p* < 0.01) number of viral genome copies in the spleen of both the vaccinated and nonvaccinated WLH group from 2 dpi onwards can be explained by the fact that after massive virus replication in the bursa of Fabricius, viremia develops, resulting in secondary virus replication in the spleen, thymus, cecal tonsils, and kidneys at 2–8 dpi [53].

Significantly, a high viral load in cecal tonsils of nonvaccinated WLH was observed at 1, 4, and 5 dpi, which can be attributed to viral replication in gut-associated lymphoid tissue after initial oral infection (*p* < 0.001, Table 5). Viral loads remained non-significant in cecal tonsils of groups other than NWG during the entire study duration. Persistent copies of the viral genome in the thymus and histopathological lesions were observed in both nonvaccinated and vaccinated WLH in our study but not in the thymus of vaccinated and nonvaccinated Aseel birds. Earlier studies [54] showed marked atrophy with severe apoptosis of thymocytes in the acute stage of IBDV infection. The differences in peak levels of viral loads in different organs of chickens in the four experimental groups in the present study might be because of their vaccination statuses or the different replication efficiency of the virus in different breeds.

**Figure 5:**
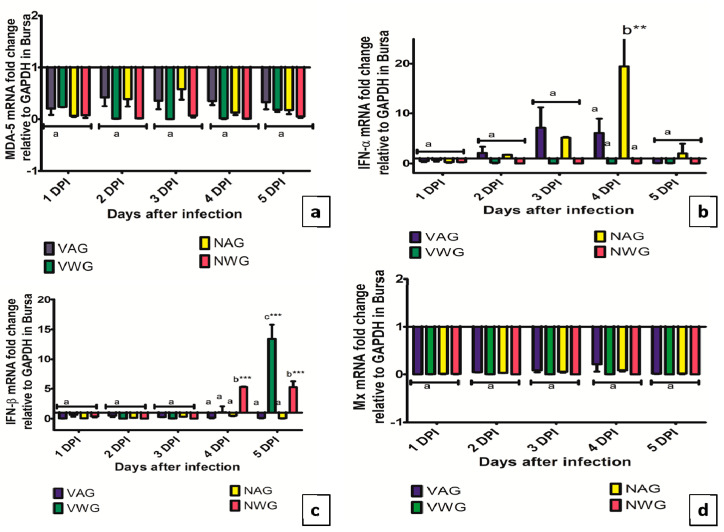
Expression of (**a**) MDA-5 (**b**) IFN-α (**c**) IFN-β (**d**) Mx. genes in Bursa of vaccinated and Non-vaccinated Aseel and White Leghorn chicken infected with very virulent IBDV. The groups were as follows: VAG-Vaccinated Aseel Group; VWG-Vaccinated White Leghorne group; NAG-Non-vaccinated Aseel Group; NWG- Non-vaccinated White Leghorn Group. Error bars represent standard error of the mean. Relative changes in the expression of genes were quantified by real-time PCR by comparison with expression levels of the housekeeping gene GAPDH and expressed as the fold-change. The value of 1.00 was kept as the control and the fold change values which were above the control values were considered as significant. * significant difference comparing VAG with VWG and NAG with NWG chickens of the same line at the same point where *p* < 0.01 **, *p* < 0.001 ***. (**a**–**c**): Experimental groups sharing common lowercase superscript letters are statistically non-significant for each observation day.

The expression of MDA-5 in the bursa of Fabricius is shown in Figure 5a. The expression of the MDA-5 gene in the bursa of Fabricius of both two breeds had shown downregulation following infection with vvIBDV throughout the study period. The expression data for IFN-α and IFN-β in the bursa of Fabricius were shown in Figure 5b,c, respectively. The expression of IFN- α gene in the bursa of Fabricius was significantly higher (19-fold) in NAG than that in the NWG at 4 dpi (*p* < 0.01). The expression of IFN-β revealed a five-fold rise in the bursa of Fabricius of NWG at 4th and 5th DPI (*p* < 0.001). At 5 dpi, VWG has significantly higher IFN-β mRNA expression in bursa compared to the VAG (*p* < 0.001).

**Figure 6:**
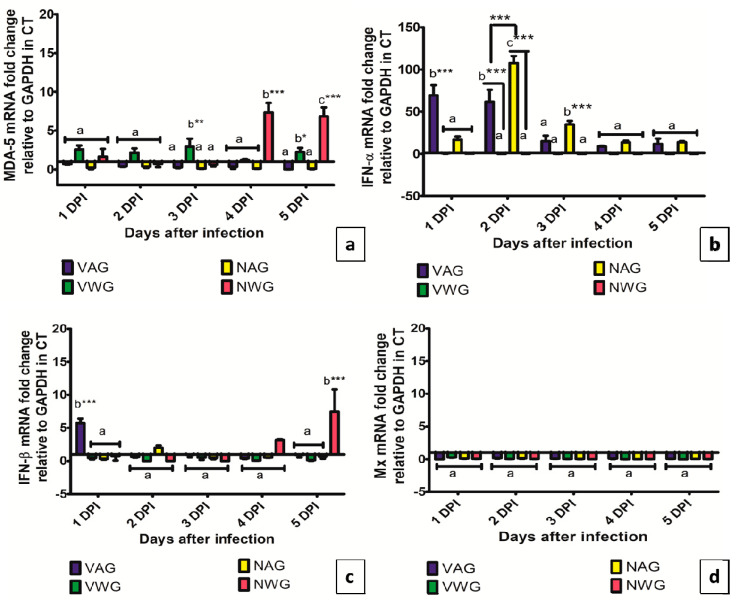
Expression of (**a**) MDA-5 (**b**) IFN-α (**c**) IFN-β (**d**) Mx. genes in Caecal tonsil of vaccinated and Non-vaccinated Aseel and White Leghorn chicken infected with very virulent IBDV. The groups were as follows: VAG-Vaccinated Aseel Group; VWG-Vaccinated White Leghorn group; NAG-Non-vaccinated Aseel Group; NWG- Non-vaccinated White Leghorn Group. Error bars represent standard error of the mean. Relative changes in the expression of genes were quantified by real-time PCR by comparison with expression levels of the housekeeping gene GAPDH and expressed as the fold-change. The value of 1.00 was kept as the control and the fold change values which were above the control values were considered as significant. * significant difference comparing VAG with VWG and NAG with NWG chickens of the same line at the same point where *p* < 0.05 *, *p* < 0.01 **, *p* < 0.001 ***. (**a**–**c**): Experimental groups sharing common lowercase superscript letters are statistically non-significant for each observation day.

The expression of MDA-5 gene in caecal tonsil is shown in Figure 6a. At 4 and dpi, the NWG has significantly higher MDA-5 mRNA expression in CT compared to the NAG (*p* < 0.001). Meanwhile, the expression of this gene in VWG has significantly higher compared to the VAG at 3 dpi (*p* < 0.01). The expression of IFN-α and IFN-β in CT was shown in Figure 6b,c, respectively. A 70- and 80- fold rise in the expression of IFN-α in CT of VAG was observed at 1 and 2 dpi, respectively, when compared to that in CT of the VWG (*p* < 0.001), which later on sharply declined to non-significant level at 3, 4, and 5 dpi. Similarly, a 100- and 35-fold increase in IFN-α expression was noticed in NAG compared to NWG at 2 and 3 dpi respectively. The expression of IFN-β gene increased moderately in CT of VAG during 1 dpi (*p* < 0.001) and in CT of NWG at 5 dpi (*p* < 0.001). No significant difference was observed in the expression of Mx gene in all the experimental groups during the study period.

**Figure 7:**
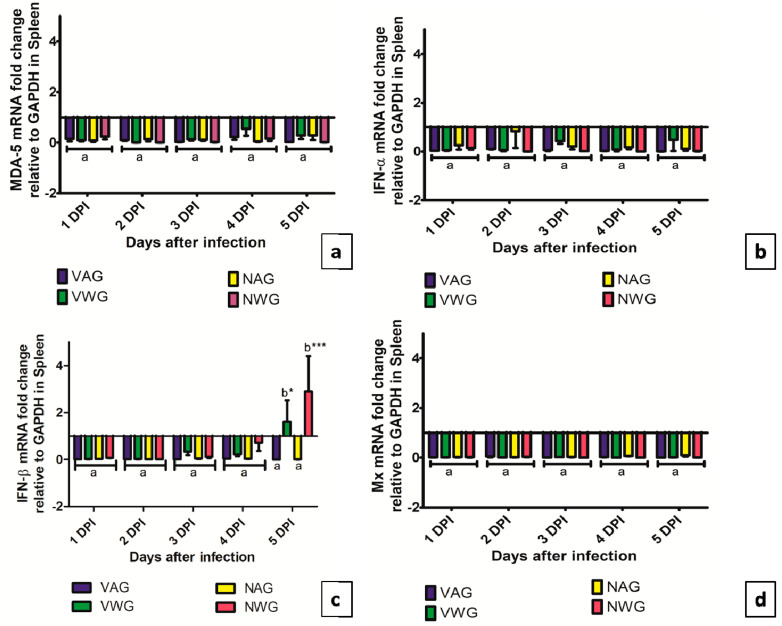
Expression of (**a**) MDA-5 (**b**) IFN-α (**c**) IFN-β (**d**) Mx genes in spleen of vaccinated and Non-vaccinated Aseel and White Leghorn chicken infected with very virulent IBDV. The groups were as follows: VAG-Vaccinated Aseel Group; VWG-Vaccinated WhiteLeghorn group; NAG-Non-vaccinated Aseel Group; NWG- Non-vaccinated WhiteLeghorn Group. Error bars represent standard error of the mean. Relative changes in the expression of genes were quantified by real-time PCR by comparison with expression levels of the housekeeping gene GAPDH and expressed as the fold-change. The value of 1.00 was kept as the control and the fold change values which were above the control values were considered as significant. * significant difference comparing VAG with VWG and NAG with NWG chickens of the same line at the same point where *p* < 0.05 *, *p* < 0.001 ***. a,b: Experimental groups sharing common lowercase superscript letters are statistically non-significant for each observation day.

The expression of MDA-5, IFN- α and Mx gene in spleens are shown in Figure 7a,b,d. The expression of all three genes showed down-regulation in the spleen of all groups of birds. A significant increase in IFN- β expression was observed at 5 dpi in VWG and NWG groups (*p* < 0.05 and *p* < 0.001) respectively as shown in Figure 7c.

**Figure 8:**
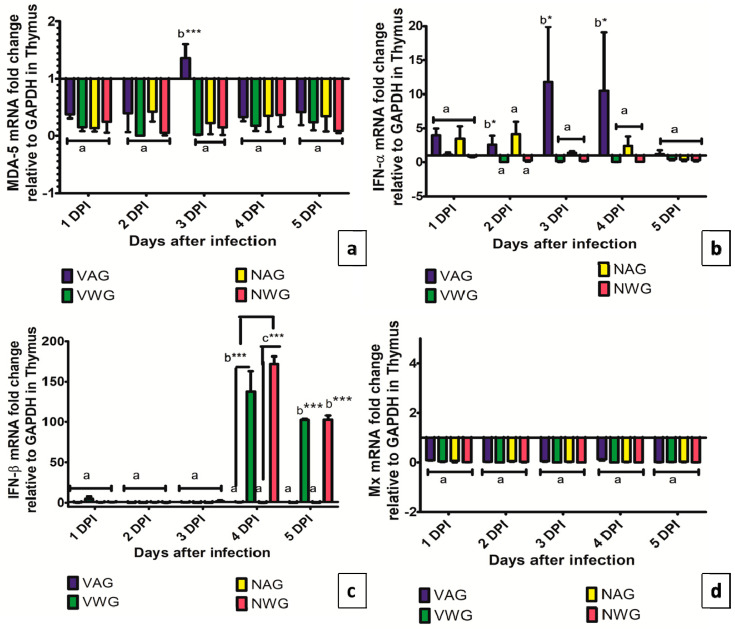
Expression of (**a**) MDA-5 (**b**) IFN-α (**c**) IFN-β (**d**) Mx. genes in thymus of vaccinated and non-vaccinated Aseel and White Leghorn chicken infected with very virulent IBDV. The groups were as follows: VAG-Vaccinated Aseel Group; VWG-Vaccinated WhiteLeghorn group; NAG-Non-vaccinated Aseel Group; NWG- Non-vaccinated WhiteLeghorn Group. Error bars represent standard error of the mean. Relative changes in the expression of genes were quantified by real-time PCR by comparison with expression levels of the housekeeping gene GAPDH and expressed as the fold-change. The value of 1.00 was kept as the control and the fold change values which were above the control values were considered as significant. * significant difference comparing VAG with VWG and NAG with NWG chickens of the same line at the same point where *p* < 0.05 *, *p* < 0.001 ***. (**a**–**c**): Experimental groups sharing common lowercase superscript letters are statistically non-significant for each observation day.

The expression of MDA-5 in the thymus is shown in Figure 8a. vvIBDV infection caused minor but significant upregulation of expression of the MDA-5 gene in the thymus of VAG at 3 dpi (*p* < 0.001). The expression data for IFN-α and IFN-β in the thymus are shown in Figure 8b,c, respectively. The expression of IFN-α in the thymus of NAG has an increased tendency at 2 and 3 dpi, followed by a decrease to nonsignificant level by 5 dpi (*p* < 0.05). The expression of IFN- β in the thymus of NWG was 170- and 100-fold higher when compared to that in the thymus of NAG (*p* < 0.001). In addition, vvIBDV infection caused the increase of expression (130- and 100-fold) of IFN-β in the VWG at 4 and 5 dpi respectively (*p* < 0.001). None of the groups showed upregulation in the expression of the Mx gene in the thymus of vvIBDV infected birds.

Interferon (IFN) responses, mediated by IFN-stimulated genes (ISGs), are the most profound innate immune responses against viruses. The transcription of the antiviral cytokines chMDA5, chIFN- α, chIFN-β, and chMx in different lymphoid organs were evaluated after normalization using GAPDH as reference genes.

In a previous study, chicken MDA-5 was found to be ubiquitously expressed in different tissues, with the highest expression in the intestines, followed by cecal tonsils and the thymus [34]. Oral administration of the virus could be responsible for inducing increased expression of MDA-5 in cecal tonsils. Differential activity of type I interferons in chickens has been suggested by previous studies [55,56].

In the present study, no increase in IFN-α could be observed in any organ or at any time point in WLH birds. In contrast, IFN-α increased beginning at 2 dpi in the bursae and thymuses of chickens in both Aseel groups. A 100-fold increase in IFN-α was noted in cecal tonsils on 2 dpi in non-vaccinated Aseel chickens, and a 70-fold increase was noted on one day in vaccinated Aseel chickens. Similar upregulation of IFN-α was seen in resistant lines of chicken challenged with IBDV [57]. A lack of IFN-α early in infection is thought to offer a chance for the virus for establishing infection [58]. In the IBDV infected commercial chickens, interferon (IFN)-α and β were significantly increased compared with the indigenous chicken breed [38]. An increase in IFN-α in the cecal tonsils of Aseel could be the result of the induction of receptors other than MDA-5. Chicken IFN-α and IFN-β showed different induction potencies for various sets of interferon-stimulated genes (ISGs), and the stronger antiviral activity of chicken IFN-α than IFN-β is credited to higher expression levels of downstream antiviral ISGs [59].

Relative expression of INF-β was increased on 4 and 5 dpi in all four organs of WLH. A similar increase in IFN-β was found in the bursae of SPF chicken on 3, 5, and 7 dpi when inoculated with classical and variant strains of IBDV [58]. Many researchers have shown increased IFN-β expression through upregulation of MDA-5 in response to Poly I:C in the cytoplasm [9,34,60,61]. A 10-fold increase in IFN-β in the cecal tonsils of VWG might have resulted in MDA-5 upregulation. However, a great increase in IFN-β in the thymuses of WLH on 4 and 5 dpi could not be attributed to the induction of MDA-5, because there was no change in MDA-5 expression in this organ. Some other pattern recognition receptors (PRRs) such as TLR-3 might be responsible for the high levels of IFN-β expression. In the case of Aseels, an increase in IFN-β was noted only in cecal tonsils on 1 and 2 dpi in nonvaccinated and vaccinated Aseel groups, respectively.

The Mx proteins are key components and its coding protein had been shown to be induced by interferon (IFN) namely IFN-α and IFN-β to inhibit the replication of RNA virus. In Atlantic salmon, replication of the infectious pancreatic necrotic virus, the prototype of *Birnaviridae* was prevented by type I IFNs, because of the expression of Mx proteins [62]. In our study, there was no significant increase in the expression of the Mx gene in any group or breed, indicating that the Mx gene played no significant role in the pathology observed in the selected genetic lines in the present study. Moreover, recent studies have demonstrated a highly polymorphic nature of the Mx gene and multiple Mx alleles exist in chickens [63]. Some reports have suggested only a specific variant confers antiviral activity [64]. Mx is a large protein with multiple functional domains. “Functional” variants of the Mx gene in different chicken lines have been found to exist, which might cause resistance or susceptibility to viral infections [65].

Earlier studies have shown that local innate effectors such as TLR-3 and IL-15 are differentially upregulated in the spleens of Aseel chicks at 3 dpi by vvIBDV [38]. The present study indicates that in an indigenous breed such as the Aseel, lymphoid tissues such as the spleen, thymus, and cecal tonsils show minimal to no lesions, as well as undetectable viral loads, following challenge with vvIBDV. The bursa also showed less severe histological lesions than those in commercial WLH, despite high viral loads. This indicates that certain innate factors other than MDA-5 receptors and the Mx protein might play a role in the resistance of this breed. These other innate immune factors should be further explored to determine the mechanism underlying the reduced susceptibility of Aseel to vvIBDV. Genetic resistance is known to be associated with several outcomes of IBDV infection, including reduced severity of IBDV-induced histological lesions in the thymus and bursa, as well as reduced mortality [66].

## 4. Conclusions

To summarize, the virulence of the IBDV is determined by its ability to cause death, which, in turn is determined by the ability of the virus to infect lymphoid organs, including the bursa. The vvIBDV strain used in this study induced lesions in lymphoid organs such as the thymus, spleen, and cecal tonsils, in addition to the bursa, in the case of WLH. The bursa was the only organ showing significant changes in Aseel, indicating that some innate factor(s) in this breed is or are responsible for this difference in pathology. Therefore, innate immune response genes other than MDA-5 and Mx should be explored to better understand this difference in pathology exhibited by Aseel and WLH. Knowledge of chicken IFNs and their antiviral activity is scarce; hence, future research aimed at understanding the molecular mechanism of IFN-α induction and associated antiviral factors is warranted. IFN-α has been shown to be related to genetic differences linked with susceptibility to different viruses and its expression levels varied after vvIBDV infection in different chicken lines. Expression of the PRR MDA-5 was limited to cecal tonsils in WLH, whereas no expression in lymphoid organs of Aseel birds was observed here. Additional studies should be directed at characterizing the most effective PRR signal transducer for vvIBDV, specifically in the apparent absence of essential components of IFN pathways, such as RIG-I, that potentiates a strong innate immune response in native chickens.

## Figures and Tables

**Table 1:** Details of primer sequences used for Quantitative Real-Time PCR.

Gene	Primer Sequence (5′ → 3′)	Product Size (bp)	Slope *	Amplification Factor	PCR Efficiency
IFN-α [33]	ATCCTGCTGCTCACGCTCCTTCT GGTGTTGCTGGTGTCCAGGATG	113	−3.295	2.01	101.14
IFN-β [34]	ACCAGGATGCCAACTTCTCTTGGA ATGGCTGCTTGCTTCTTGTCCTTG	145	−3.673	1.87	87.18
M_X_ [35]	GCACACACCCAACTGTCAGCGA CCCATGTCCGAAACTCTCTGCGG	156	−3.182	2.06	106.19
MDA5 [34]	TGCAAGGGAGCTGTTGAGCAGAAT GGCTTTCATGTTGGGTTTCTCGCA	89	−3.251	2.03	103.05
GAPDH [35]	ATCAAGAGGGTAGTGAAGGCTGCT TCAAAGGTGGAGGAATGGCTGTCA	116	−3.441	1.95	95.26
vvIBDVVP2 gene [36]	AGATAACCCAGCCAATCAC CACTCTTTCGTAAGCTACTAGTG	173	−3.239	2.036	103.5

* Slope at 95% confidence level, R^2^: 0.9978.

**Table 2:** Depicting the semi-quantitative assessment of the severity of disease based on gross lesions in lymphoid organs among the challenged and control chicken of various groups.

Group	White Leghorn			Aseel		
	VWG	NWG	CWG	VAG	NAG	CAG
1 dpi	+	+	-	-	-	-
2 dpi	+	++	-	-	++	-
3 dpi	-	++	-	-	++	-
4 dpi	+	+++	-	-	+	-
5 dpi	+	+++	-	-	+	-

Score/ Grade: - for no gross lesions and appearing normal; + for bursa slightly enlarged and edematous; ++ for edematous, enlarged or hemorrhagic bursa with changes in any of the organs (hemorrhages in thymus/ necrotic foci in spleen/hemorrhage in caecal tonsils) and +++ for gross changes in all the lymphoid organs. VWG: Vaccinated challenged WLH group; NWG: Nonvaccinated challenged WLH group; CWG: WLH control group; NAG: Nonvaccinated challenged Aseel group; VAG: Vaccinated challenged Aseel group; CAG: Aseel control group.

**Table 3:** Depicting the histopathological lesion scores (Mean ± SE) in various lymphoid organs in different groups at different intervals of time.

Organ	Groups	Day 1	Day 2	Day 3	Day 4	Day 5
Bursa	VWG	2.00 ± 0.0 ^c^	2.333 ± 0.33 ^bc^	1.667 ± 0.33 ^b^	2.333 ± 0.33 ^b^	2.333 ± 0.33 ^b^
	NWG	2.00 ± 0.57 ^c^	2.667 ± 0.33 ^c^	3.00 ± 0.57 ^c^	3.333 ± 0.33 ^c^	4.333 ± 0.0 ^c^
	CWG	0.0 ± 0.0 ^a^	0.00 ± 0.0 ^a^	0.00 ± 0.0 ^a^	0.00 ± 0.0 ^a^	0.00 ± 0.0 ^a^
	VAG	0.00 ± 0.0 ^a^	0.3333 ± 0.33 ^a^	0.3333 ± 0.33 ^a^	0.6667 ± 0.33 ^a^	0.3333 ± 0.33 ^a^
	NAG	1.00 ± 0.0 ^b^	1.667 ± 0.33 ^b^	2.667 ± 0.33 ^c^	3.333 ± 0.33 ^c^	2.00 ± 0.33 ^b^
	CAG	0.00 ± 0.0 ^a^	0.00 ± 0.0 ^a^	0.00 ± 0.0 ^a^	0.00 ± 0.0 ^a^	0.00 ± 0.0 ^a^
Spleen	VWG	0.3333 ± 0.33 ^a^	1.667 ± 0.33 ^b^	1.00 ± 0.57 ^b^	0.6667 ± 0.33 ^a^	1.00 ± 0.33 ^b^
	NWG	0.3333 ± 0.33 ^a^	2.667 ± 0.33 ^c^	2.667 ± 0.33 ^c^	2.333 ± 0.33 ^b^	2.00 ± 0.33 ^c^
	CWG	0.00 ± 0.0 ^a^	0.0 ± 0.0 ^a^	0.0 ± 0.0 ^a^	0.0 ± 0.0 ^a^	0.0 ± 0.0 ^a^
	VAG	0.00 ± 0.0 ^a^	0.6667 ± 0.33 ^a^	0.3333 ± 0.33 ^a^	0.0 ± 0.0 ^a^	0.0 ± 0.0 ^a^
	NAG	0.6667 ± 0.33 ^a^	0.6667 ± 0.33 ^a^	1.333 ± 1.33 ^b^	0.0 ± 0.0 ^a^	0.0 ± 0.0 ^a^
	CAG	0.00 ± 0.00 ^a^	0.0 ± 0.0 ^a^	0.0 ± 0.0 ^a^	0.0 ± 0.0 ^a^	0.0 ± 0.0 ^a^
Thymus	VWG	0.6667 ± 0.33 ^a^	0.6667 ± 0.33 ^a^	1.333 ± 0.33 ^b^	1.667 ± 0.33 ^b^	1.00 ± 0.00 ^b^
	NWG	0.6667 ± 0.33 ^a^	1.00 ± 0.00 ^b^	1.667 ± 0.33 ^b^	2.333 ± 0.33 ^b^	2.667 ± 0.33 ^c^
	CWG	0.0 ± 0.0 ^a^	0.0 ± 0.0 ^a^	0.0 ± 0.0 ^a^	0.0 ± 0.0 ^a^	0.0 ± 0.0 ^a^
	VAG	0.0 ± 0.0 ^a^	0.0 ± 0.0 ^a^	0.0 ± 0.0 ^a^	0.0 ± 0.0 ^a^	0.0 ± 0.0 ^a^
	NAG	0.6667 ± 0.33 ^a^	0.3333 ± 0.33 ^a^	0.0 ± 0.0 ^a^	0.0 ± 0.0 ^a^	0.0 ± 0.0 ^a^
	CAG	0.0 ± 0.0 ^a^	0.0 ± 0.0 ^a^	0.0 ± 0.0 ^a^	0.0 ± 0.0 ^a^	0.0 ± 0.0 ^a^
Caecal tonsils	VWG	0.6667 ± 0.33 ^a^	1.333 ± 0.33 ^b^	1.00 ± 0.00 ^b^	1.667 ± 0.33 ^b^	2.00 ± 0.00 ^b^
	NWG	1.00 ± 0.57 ^b^	2.333 ± 0.33 ^c^	2.333 ± 0.33 ^c^	1.667 ± 0.33 ^b^	1.667 ± 0.33 ^b^
	CWG	0.0 ± 0.0 ^a^	0.0 ± 0.0 ^a^	0.0 ± 0.0 ^a^	0.0 ± 0.0 ^a^	0.0 ± 0.0 ^a^
	VAG	0.3333 ± 0.33 ^a^	0.3333 ± 0.33 ^a^	0.0 ± 0.0 ^a^	0.3333 ± 0.33 ^a^	0.0 ± 0.0 ^a^
	NAG	1.667 ± 0.33 ^b^	1.667 ± 0.33 ^bc^	1.667 ± 0.33 ^bc^	0.3333 ± 0.33 ^a^	0.0 ± 0.0 ^a^
	CAG	0.0 ± 0.0 ^a^	0.0 ± 0.0 ^a^	0.0 ± 0.0 ^a^	0.0 ± 0.0 ^a^	0.0 ± 0.0 ^a^

(**a–c**): Different lowercase superscript letters within a column for each lymphoid organ indicate significance difference (*p* < 0.05); NWG: Nonvaccinated challenged WLH group; VWG: Vaccinated challenged WLH group; CWG: WLH control group; NAG: Nonvaccinated challenged Aseels group; VAG: Vaccinated challenged Aseels group; CAG: Aseel controls group.

**Table 4:** Immunohistochemical scoring of bursal sections in different groups of WLH and Aseel birds at different intervals of time.

	White Leghorn			Aseel		
Group	VWG	NWG	CWG	VAG	NAG	CAG
1 dpi	+	++	-	-	++	-
2 dpi	+++	+++	-	+	+++	-
3 dpi	+++	+++	-	+	+++	-
4 dpi	+++	+++	-	++	+++	-
5 dpi	+++	+++	-	++	+++	-

Score/ Grade: - for no staining, + for minimal staining (<10% of cells present), ++ for moderate staining (>10% and <30% of cells present), +++ for intense staining (>30% of cells present); NWG: Nonvaccinated challenged WLH group; VWG: Vaccinated challenged WLH group; CWG: WLH control group; NAG: Nonvaccinated challenged Aseel group; VAG: Vaccinated challenged Aseel group; CAG: Aseel controls group.

**Table 5:** Viral copy number (Mean± SEM) in various organs of challenged birds.

Organ	DPI	VAG	NAG	VWG	NWG
Bursa	1 DPI	1289.3 ± 12.7 ^a^	1469.8 ± 57.3 ^a^	948.9 ± 53.4 ^a^	778.8 ± 118.1 ^a^
	2 DPI	0.0 ± 0.0 ^a^	23412.9 ± 368.5 ^d^	5525.2 ± 154.1 ^c^	1459.5 ± 111.7 ^b^
	3 DPI	0.0 ± 0.0 ^a^	0.0 ± 0.0 ^a^	8615.1 ± 301.8 ^c^	1492.5 ± 83.4 ^b^
	4 DPI	0.0 ± 0.0 ^a^	0.0 ± 0.0 ^a^	5282.1 ± 231.1 ^b^	4999.8 ± 236.7 ^b^
	5 DPI	0.0 ± 0.0 ^a^	0.0 ± 0.0 ^a^	1786.8 ± 75.6 ^b^	8937.8 ± 427 ^c^
CT	1 DPI	0.0 ± 0.0 ^a^	0.0 ± 0.0 ^a^	0.0 ± 0.0 ^a^	12405.3 ± 664.4 ^b^
	2 DPI	0.0 ± 0.0 ^a^	0.0 ± 0.0 ^a^	625.7 ± 24.2 ^a^	1116.0 ± 118.3 ^a^
	3 DPI	0.0 ± 0.0 ^a^	868.4 ± 41.5 ^a^	1114.7 ± 93.9 ^a^	1170.3 ± 213.9 ^a^
	4 DPI	0.0 ± 0.0 ^a^	1030.3 ± 64.1 ^a^	1064.0 ± 62.5 ^a^	5092.7 ± 1280.5 ^b^
	5 DPI	0.0 ± 0.0 ^a^	1463.9 ± 58.1 ^a^	1168.7 ± 52.4 ^a^	4665.0 ± 1062 ^b^
Spleen	1 DPI	0.0 ± 0.0 ^a^	0.0 ± 0.0 ^a^	0.0 ± 0.0 ^a^	0.0 ± 0.0 ^a^
	2 DPI	0.0 ± 0.0 ^a^	0.0 ± 0.0 ^a^	5000.7 ± 265 ^b^	4500.7 ± 331.7 ^b^
	3 DPI	0.0 ± 0.0 ^a^	0.0 ± 0.0 ^a^	1065.3 ± 133.5 ^b^	7836.0 ± 318.9 ^c^
	4 DPI	0.0 ± 0.0 ^a^	0.0 ± 0.0 ^a^	1282.7 ± 97.1 ^b^	7984.0 ± 544.7 ^c^
	5 DPI	0.0 ± 0.0 ^a^	0.0 ± 0.0 ^a^	1834.3 ± 482.9 ^b^	7633.0 ± 380 ^c^
Thymus	1 DPI	1428.6 ± 65.7 ^a^	1459.1 ± 46.2 ^a^	0.0 ± 0.0 ^b^	1406.3 ± 85.8 ^a^
	2 DPI	0.0 ± 0.0 ^a^	0.0 ± 0.0 ^a^	28369.0 ± 863.8 ^c^	1301.7 ± 179.9 ^b^
	3 DPI	0.0 ± 0.0 ^a^	0.0 ± 0.0 ^a^	2477.3 ± 207.3 ^b^	1573.7 ± 65.8 ^b^
	4 DPI	0.0 ± 0.0 ^a^	0.0 ± 0.0 ^a^	1160.3 ± 60.9 ^b^	1684.7 ± 235.3 ^b^
	5 DPI	0.0 ± 0.0 ^a^	0.0 ± 0.0 ^a^	6070.7 ± 237.7 ^b^	14470.0 ± 559.5 ^c^

(**a**–**d**): Different lowercase superscript letters between experimental groups for each observation day indicate significance difference (*p* < 0.05); Values below limit of detection (LOD) were represented by 0.0. VAG- Vaccinated Aseel Group; NAG- Nonvaccinated Aseel Group VWG- Vaccinated WLH Group; NWG- Nonvaccinated WLH Group CT- Caecal tonsils.
